# Supplementary material for: Early access to physiotherapy for infants with cerebral palsy: A retrospective chart review
Source: PLoS One. 2021 Jun 25;16(6):e0253846. doi: 10.1371/journal.pone.0253846 (PMC8232431; doi:10.1371/journal.pone.0253846)
Supplement: S1 Table — (DOCX) [file pone.0253846.s001.docx]

| **S1 Table.** Template for data collection. |
| --- |
|  |
|  |
