# Supplementary material for: Early access to physiotherapy for infants with cerebral palsy: A retrospective chart review
Source: PLoS One. 2021 Jun 25;16(6):e0253846. doi: 10.1371/journal.pone.0253846 (PMC8232431; doi:10.1371/journal.pone.0253846)
Supplement: Attachment — Submitted filename: PlosOne.pdf [file pone.0253846.s002.pdf]

The authors retrospectively reviewed patient data from two health care systems in a county in Sweden in the aim to assess whether children with cerebral palsy (CP) have equal access to timely physiotherapy. They concluded from the data that Children with CP have unequal access to physiotherapy. The authors emphasized the importance of timely access of physiotherapy to children with CP. The data and results might be helpful for the improvement of local health policy for children with CP.

The authors provided detailed descriptive results of data. However, the description of data collection is not detailed. Several aspects of information as the following should be described:

(1) as a retrospective review, do you have a data collection guidance or standard, which is like on what data to be reviewed , how to collect data, and data quality control, before the formal data review? if so, please provide a data collection flow chart.

(2) as described in the manuscript, the first author reviewed most of the data from two different health care systems, is there the possibility of any potential bias on reviewing data from two different health care systems mostly by one researcher, has any potential bias been considered to be controlled before formal data review, has any preventive method been used to control the bias?

(3) the authors did not mention any data missing in the manuscript. How has data missing been handled if you did have the cases of missing data?

As to statistical analysis, the authors used Kruskal-Wallis H Test but without providing any justification for the selection of this method.

As to results, the authors found that both referral source and number of risk factors have significant influences to the first visit to physiotherapy (see Table 2), and concluded that “children referred from the child health services have the most delayed access” (see abstract conclusion). A likely scenario is that number of risk factors dominates the decision of the first visit to physiotherapy but children with CP from the child health services have the fewer number of risk factors compared to other referral sources, and the fewer number of risk factors caused the delay of referral of children with CP to physiotherapy by the child health services. If the number of risk factors was controlled, is the conclusion “children referred from the child health services have the most delayed access” still correct?
