# Supplementary material for: Early access to physiotherapy for infants with cerebral palsy: A retrospective chart review
Source: PLoS One. 2021 Jun 25;16(6):e0253846. doi: 10.1371/journal.pone.0253846 (PMC8232431; doi:10.1371/journal.pone.0253846)
Supplement: Attachment — Submitted filename: Respons to reviewers.docx [file pone.0253846.s004.docx]

Uppsala, Sweden, 4^th^ of June 2021

Response to reviewers comments regarding manuscript PONE-D-20-28394R2

We would again like to thank the reviewers for their valuable and constructive feedback! Below you find our response to the queries. We have highlighted the changes in the manuscript in yellow. Added text is highlighted, while removed text appears as **strikethrough.**

**Journal Requirements**

(1) Please review your reference list.

We have reviewed the references and it is complete and correct. Small changes have been made in the text. Additionally, we have replaced reference 6, with a recently published paper (reference 8).

Removed

Reference 6. Morgan C, Darrah J, Gordon AM, Harbourne R, Spittle A, Johnson R, et al. Effectiveness of motor interventions in infants with cerebral palsy: a systematic review. Dev Med Child Neurol. 2016;58: 900–909. doi:10.1111/dmcn.13105.

Replaced

Reference 8. Morgan C, Fetters L, Adde L, Badawi N, Bancale A, Boyd RN, et al. Early intervention for children aged 0 to 2 years with or at high risk of cerebral palsy: international clinical practice guideline based on systematic reviews. JAMA Pediatr. 2021. doi: 10.1001/jamapediatrics.2021.0878.

**Review comments to the Author**

**Reviewer #1**

(1) The authors have revised the manuscript thoroughly, and overall revisions are clear and logical. A main point I still have is on the regression analysis. Firstly, the authors did not state the regression models they used are linear or nonlinear. Secondly, the names of regressions the authors used, univariate regression and multivariate regression, are confusing. In both regression analysis, there is only one outcome variable (age at the first visit to a physiotherapist), thus the regressions should be named as simple (linear, I assume) regression for that with only one predictor and multiple (linear, I assume) regression for that with more than one predictors. Thirdly, as to the multivariate regression, the authors did not include an interaction item on referral source * number of risk factors. As mentioned before, I think there may exist interaction effect between referral source and number of risk factors. I am interested in that if the interaction item on referral source * number of risk factors was added in the multivariate regression, how would the results be?

As reviewer #1 assumed, we have used a linear regression model. We have added this to the text. To further clarify our analysis, we have changed the wording from univariate and multivariate to unadjusted and adjusted regression models. For the third point, we will start by thanking the reviewer! This was important and has added valuable information about our sample and our results. We have included the interaction item in the regression analysis and added a figure to illustrate the interaction between referral source and number of risk factors. Text addressing these results have be added where appropriate. Additionally, we have changed the order of the variable “referral source”, so that the NICU graduates are used as the reference.

Sincerely,

Kine Johansen

Physiotherapist specialised in paediatrics, PhD

Corresponding author

Department of Women’s and Children’s Health
